# Women's Work in South Africa

**Published:** 1893-09-02

**Authors:** 


### Women's Work in South Africa.

Africa is the real new world of our day, and has not fully outgrown the inarticulate stage of youth. It has not yet reached the point of active and original work in art and science; its gold and diamonds are in the rough, but one voice from beyond the equator has made itself heard in the literature of the English-speaking world. Olive Schreiner, who utters the impersonal and far-reaching altruism of a George Eliot with the fervid passion of a Charlotte Bronte, is a unique growth, independent of her surroundings even when she is most affected by them, poet and seer on the boundless rolling veldt, as she would have been poet and seer had she dwelt on misty hill side, cloistral valley, or throbbing busy town. But life precedes art, and South Africa has a record of earnest women who are working by the side of the men who are redeeming the land from heathenism, barbarism, [and desolation; or, in still more womanly fashion, follow in their footsteps to heal the wounded and save the erring who have fallen by the way.

First and most numerous come the missionaries. Times are changed since the wives of Moffatt and Livingstone went with their husbands into the wilderness, and every church has now its mission, with a band of women workers; but with increased advantages there has come the acceptance of increased responsibility, and the development of new fields of labour in educational and philanthropic work. Thus, at Cape Town, the Sisters belonging to the English Church Mission have a home for destitute children, a day school, a house of mercy, and a Middle-class School for Girls. They have also the nursing at the public hospital, the New Somerset Hospital as it is called, to distinguish it from the old Somerset Hospital, which is really an almshouse. As nurses the Sisters, who are members of St. Margaret's Sisterhood, London, do admirable work, although they complain of a lack of sympathy on the part of some of the officials. Nursing by a religious sisterhood is always likely to arouse prejudice in the minds of those who suspect an inclination on the part of the sisters to proselytize —a prejudice which is certain to die away in face of competent and conscientious work.

In Graham's Town, Port Elizabeth, and King William's Town, there are a few trained nurses at each of the hospitals. At present these are all Englishwomen, but among the duties they have taken up is the training of their Colonial sisters in nursing, wherein lies the only possibility of meeting the great and increasing demand for skilled help in sickness which is rising from hospitals and private families all through the country. Another matter of much importance which has been taken up at the Grey Hospital in King Williams' Town, which was opened about twenty years ago to counteract the fearful powers then possessed by the witch doctor, is the training of native women as nurses among the Kaffir population. This is the best way to extirpate the witch doctor; there can be no doubt that a native woman, familiar with native superstitions, habits, and prejudices, while yet she has outgrown them, will be more easily and completely successful in dealing with her sick compatriots than any European can be. Moreover, it will be possible to provide native nurses much more cheaply than the imported article, and this is a point by no means to be ignored, nor should one forget the moral effect on barbarous races, to whom women have been for ages only beasts of toil, of seeing among them women whose skill is acknowledged, and their position recognised by all.

Readers of The Hospital know what nursing on the diamond fields is like from the letters of Miss Rose Blennerhassett, descriptions marked by a cheerful wit and unfailing courage which made light of, though they could not conceal, the almost insuperable difficulties of the work. The Kimberley Hospital, which began in 1877 with eight beds, now numbers 280 beds. Two years ago a hospital was founded at Umtali, near Manicaland, under great difficulties. Trained nurses are so scarce and so highly esteemed in South Africa that people will send as far as 500 miles to secure them, and therefore it was not to be wondered at that with such a premium put on their services, those to whom money was for one reason or another a matter of the first importance, refused to go to Umtali for less than the practically prohibitive salary of £70 a year. It was then that three nurses, Misses Blennerhassett, Sleeman, and Welby, who had intended to return home, volunteered for this pioneer work, and managed to do good nursing with hardly any of the material requisites for it. It was a matter of adapting oneself to circumstances all along. Having been conveyed in a steam launch up a stagnant river, alive with crocodiles, as far as Mapanda, no further conveyance was found to be attainable, and after waiting at Mapanda for some time, they made up their minds to walk to Umtali, a walk of 270 miles, which they accomplished in thirteen days, along a road which was no road, but only a rough indication of the way to go. Sometimes it led them across a desert, sometimes it took them through a wilderness of tall grass, brown and waving, seven feet high, making a restless twilight round them which caused almost unendurable vertigo. Their bearers deserted them, and their baggage had to be left on the veldt. Worse than this, they had now no one to help them across the rivers, and were forced to wade them and risk any dangers that might chance from being soaked to the skin. And still they went bravely on. The calm courage, the cool indifference to danger possessed by these women is unconsciously crystallized in the remark of one of them, "I used to be frightened when I heard the lions roaring, now I only think them great bores."

In such places as Kimberley and mining centres generally, more than even in our own country, a great proportion of disease must be traced to intemperance, and in temperance work an increasing number of women are finding their vocation. Most prominent among them is Miss Etty Schreiner, who for a long time fought the drink fiend almost single-handed. But she was fit for the task. With her glowing eyes, her vivacious brunette countenance, her exceptional eloquence, and her grace of gesture, she possessed that combination of beauty with determination which is of no small importance when a woman has to fight a cause which is championed by men. A strong proof of her influence was once given at Kimberley, when an impassioned and persuasive appeal from her induced several publicans to bring out their casks of spirits and pour them out into the gutter. The Temperance Union of South Africa, founded by the American missionary, Mrs. Clemence Leavitt, in 1889, now numbers four hundred members, but none of these equal Miss Schreiner in prominence.

At present South Africa can boast of only one medical woman, Miss Waterson, M.D. She came to the Cape immediately after obtaining her diploma, and first undertook work at Blantyre. She was specially engaged by the trading company there, but the Mission also soon fell under her charge. At that time there was no male doctor at the Mission; the sole responsibility in all forms of accident and disease fell upon Miss Waterson. At last her health gave way, and she was forced to leave Blantrye. She went to Cape Town, where, not without a struggle against opposition and prejudice, she has succeeded in obtaining a large and successful practice. Part of this is of a remunerative character, but Miss Waterson devotes also a considerable portion of her time to the poor, and has accomplished philanthropic work of no common order in the physical relief and moral reformation of fallen women.

These women are doubtless only pioneers in a great march of progress, but the work they have done in a very few years shows that they are alive to the greatness of the needs and opportunities of South Africa, whether it is their native land or the country of their adoption.

